# Interference with lysophosphatidic acid receptor 5 ameliorates oxidized low-density lipoprotein-induced human umbilical vein endothelial cell injury by inactivating NOD-like receptor family, pyrin domain containing 3 inflammasome signaling

**DOI:** 10.1080/21655979.2021.1983975

**Published:** 2021-10-18

**Authors:** Ling Xu, Chaoxiang Xu, Xiaoxin Lin, Huiyao Lu, Yinlian Cai

**Affiliations:** Department of Cardiovascular Medicine, The Second Attached Hospital of Fujian Medical University, Quanzhou, Fujian, China

**Keywords:** Atherosclerosis, lpar5, inflammation, oxidative stress, endothelium dysfunction

## Abstract

Endothelial cell damage induced by oxidized low-density lipoprotein (ox-LDL) plays an important role in the pathogenesis of atherosclerosis (AS). We aimed to explore the effects of lysophosphatidic acid receptor 5 (LPAR5) on ox-LDL-induced damage of human umbilical vein endothelial cells (HUVECs). After HUVECs exposed to ox-LDL, LPAR5 expression was detected by RT-qPCR and western blotting. Then, LPAR5 was silenced and cell viability was determined with a CCK-8 assay. ELISA was employed to analyze the contents of inflammatory factors. The levels of oxidative stress markers were examined by kits. The expression of proteins related to endothelium function, including CD31, α-SMA, iNOS and eNOS, was evaluated with RT-qPCR and western blotting. Additionally, the effects of LPAR5 deletion on the NLRP3 inflammasome signaling in HUVECs under ox-LDL condition were assessed by determining NLRP3, caspase-1 and ASC expression. Afterward, NLRP3 agonist MSU was adopted for exploring the regulation of LPAR5 on NLRP3 inflammasome signaling in ox-LDL HUVECs injury. Results revealed that ox-LDL led to a significant upregulation in LPAR5 expression. NLRP3 knockdown enhanced cell viability, inhibited inflammation and oxidative stress in HUVECs after ox-LDL exposure. Besides, the expression of CD31 and eNOS was increased while that of α-SMA and iNOS was decreased after LPAR5 silencing. Moreover, interference with LPAR5 remarkably downregulated NLRP3, caspase-1 and ASC expression. Furthermore, MSU addition partially abrogated the inhibitory effects of LPAR5 deletion on the inflammation, oxidative stress and endothelium dysfunction of HUVECs. To conclude, we demonstrated that LPAR5 silencing alleviates ox-LDL-induced HUVECs injury by inhibiting NLRP3 inflammasome signaling.

## Introduction

Atherosclerosis (AS), a common chronic inflammatory vascular disease, is the major cause of cardiovascular disease, which is becoming a global concern due to its high prevalence [1,2]. It is usually related to severe consequences, economic burdens and high mortality rates worldwide [3]. Although the pathology of AS is complex, it is well accepted that the dysfunction of the cells that constitute the endothelium is a primary driving force in the initiation of the disease and oxidized low-density lipoprotein (ox-LDL) is a well-known exceptionally key risk factor in this process [4]. Ox-LDL can induce reactive oxygen species (ROS) overproduction, disrupt the redox balance of vascular endothelial cells, increase local inflammatory response and trigger adhesion molecule release, thereby resulting in endothelial damage. In turn, oxidative stress accelerates the oxidation of low-density lipoprotein in the blood vessel wall, which further aggravates the vascular endothelium injury [5–7]. Therefore, inhibiting ox-LDL-induced endothelial cell damage has become a promising treatment strategy to prevent AS.

Lysophosphatidic acid receptor 5 (LPAR5), the most recently found member of the LPAR family, is a G protein-coupled transmembrane receptor that play a crucial role in various disease, such as cardiovascular disease, airway disease, obesity and cancer [8]. It has been reported that lysophosphatidic acid (LPA) transactivates TGFBR1 via upregulating LPAR5 expression, which leads to inner membrane lipid retention and inducing the early onset of AS [9]. Another study has suggested that LPAR5 is closely implicated in thrombosis after atherosclerotic plaque rupture [10]. There is no research report so far on the role of LPAR5 in endothelial cell injury during the progression of AS. An increasing number of researches have validated that the activation of NOD-like receptor family, pyrin domain containing 3 (NLRP3) inflammasome signaling participates in the malignant progression of AS [11–13]. It is worthy of note that LPAR5 can activate NLRP3 inflammasome in macrophages and contribute to imiquimod-induced psoriasis-like lesions [14]. Therefore, this study focused on the role of LPAR5 in ox-LDL endothelial cell injury and the possible mechanisms related to NLRP3 inflammasome.

In the present study, human umbilical vein endothelial cells (HUVECs) stimulated by ox-LDL were used to simulate the endothelial cell injury during AS. The effects of LPAR5 on the oxidative stress, inflammation and endothelium dysfunction of HUVECs induced by ox-LDL and its regulatory effects on NLRP3 inflammasome signaling were explored. A successful study may provide a promising strategy for AS therapy.

## Materials and methods

### Cell culture and treatment

HUVECs were provided by the American Type Culture Collection (Manassas, VA, USA). Cells were cultured with Dulbecco’s modified Eagle’s medium (DMEM, Invitrogen, Carlsbad, USA) containing 10% fetal bovine serum (FBS, Gibco). HUVECs were cultured at 37°C in a humidified chamber containing 95% air and 5% CO_2._ When grown to 80% confluence, HUVECs were stimulated with 100 µg/ml ox-LDL for 24 h. Monosodium urate (MSU), a NLRP3 agonist, was used to treat HUVECs for 6 h at the concentrations of 100 μg/mL prior to ox-LDL exposure [15].

## Cell transfection

For cell transfection, small interfering RNAs (siRNA) targeting LPAR5 (si-LPAR5#1 and si-LPAR5#2) and scramble oligonucleotide (siRNA-NC) synthesized by GenePharma (Shanghai, China) were employed to silence LPAR5. HUVECs were inoculated into 6-well plates and transfection experiments were conducted using Lipofectamine 2000 reagent (Invitrogen, Carlsbad, CA) following the instructions for the kit. After transfection for 24 h, cells were collected to evaluate the transfection efficiency.

## Cell viability assay

Cell viability was tested by the cell counting kit-8 (CCK-8) kit purchased from Solarbio Biotechnology (Beijing, China). 5 × 10^3^ cell HUVECs were seeded in 96-well plates. After treatment, each well was supplemented with 10 μl of CCK-8 solution for 2 h. The optical densities at the wavelength of 450 nm were investigated using a microplate reader.

## Measurement of inflammatory factors

HUVECs were seeded into 6-well plates. After transfection and ox-LDL stimulation, the cell culture supernatant was collected, the secretion of tumor necrosis factor (TNF)-α, interleukin (IL)-1β and IL-6 were examined with enzyme-linked immunosorbent assay (ELISA) kits according to standard protocol provided by Shanghai XiTang Biotechnology (Shanghai, China). The absorbance at 450 nm was tested using a microplate reader.

## ROS production

A ROS assay kit (Beyotime, Shanghai, China) was utilized for the determination of intracellular ROS production. Briefly, HUVECs were incubated with 10 μM dichloro-dihydro-fluorescein diacetate (DCFH-DA) for 20 min in the dark. The fluorescence values were reflected by the fluorescence released using a fluorescence spectrophotometer at 490 nm excitation and 585 nm emission.

## Detection of the oxidative stress markers

The malondialdehyde (MDA) and nitric oxide (NO) contents as well as superoxide dismutase (SOD) and glutathione peroxidase (GSH-Px) activities in HUVECs were detected using the lipid peroxidation MDA assay kit, SOD activity assay kit, GSH-Px activity assay kit and NO Detection Kit, respectively, following manufacturer’s recommendations. Aforementioned kits were obtained from Nanjing Jiancheng Bioengineering Institute (Nanjing, China).

## RNA extraction and reverse transcription-quantitative polymerase chain reaction (RT-qPCR) analysis

Total RNA was extracted from HUVECs with TRIzol reagent (Invitrogen, Carlsbad, CA, USA). Prime Script™ RT reagent kit (Takara, Japan) was adopted for the synthesis of complementary DNA (cDNA). Then, cDNA amplification was performed on a PCR instrument using the SYBR Green Mastermix kit (Takara, Dalian, China) on a Prism 7700 sequence detection system (Applied Biosystems). The housekeeping gene glyceraldehyde-phosphate dehydrogenase (GAPDH) was used as an internal control. The 2^−ΔΔCq^ method was used to quantify the expression of target genes [16].

## Western blot analysis

Cells were lysed in radioimmunoprecipitation assay (RIPA) buffer to obtain the total protein. Protein concentrations in the lysates were quantified with a BCA method (Beyotime, Shanghai, China). Equal amounts of proteins were separated by loading on a 10% gel for sodium dodecyl sulfate-polyacrylamide gel electrophoresis (SDS-PAGE), followed by electrophoresed and transferred to a polyvinylidene fluoride membrane (PVDF). 5% skim milk was employed to block the nonspecific proteins on the membranes. These blots were then probed with primary antibodies at 4°C overnight. The secondary antibody was added the next day and incubated at room temperature for 1 h. Protein bands were visualized by an Odyssey Infrared Imaging Scanner (LI-COR Biosciences). Band densities of target proteins were normalized to that of GAPDH and quantified using Image J software.

## Statistical analysis

Data were expressed as the mean ± standard deviation of three independent experiments. Analysis was conducted by GraphPad Prism 8.0. The comparison employed student’s t-test for two groups and one-way analysis of variance (ANOVA) followed by Tukey’s post hoc test for multiple groups. *P* value <0.05 was considered significant.

## Results

### Ox-LDL stimulation upregulates LPAR5 expression and LPAR5 silencing alleviates ox-LDL-induced inflammation and oxidative stress of HUVECs

**Figure 1.**
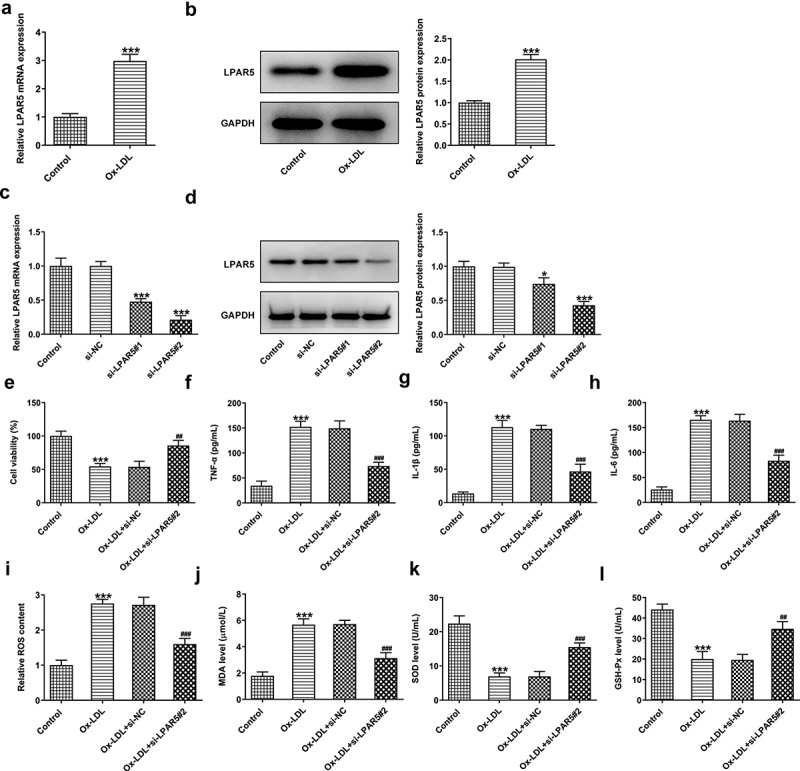
Ox-LDL stimulation enhanced LPAR5 expression and LPAR5 silencing alleviated ox-LDL-induced inflammation and oxidative stress of HUVECs. The mRNA and protein expression of LPAR5 in HUVECs under ox-LDL exposed condition was analyzed by (a) RT-qPCR and (b) western blot analysis. ***P < 0.001 vs. control. Assessment of LPAR5 mRNA and protein expression using (c) RT-qPCR and (d) western blot assay. *P < 0.05, ***P < 0.001 vs. si-NC. (e) Cell viability was examined by means of a CCK-8 kit. Measurement of the contents of (f) TNF-α, (g) IL-1β and (h) IL-6 using ELISA kits. The contents of (i) ROS, (j) MDA and the activities of (k) SOD and (l) GSH-Px were tested with commercially available kits. ***P < 0.001 vs. control; ^##^*P* < 0.01, ^###^*P* < 0.001 vs. ox-LDL+si-NC

It has been reported that LPAR5 is closely implicated in thrombosis after atherosclerotic plaque rupture [10]. First, the expression of LPAR5 in HUVECs exposed to ox-LDL was determined with RT-qPCR and western blot analysis. As exhibited in Figure 1a-B, ox-LDL induction remarkably elevated LPAR5 mRNA and protein expression compared with the untreated control group. Then, si-LPAR5#1 or si-LPAR5#2 was transfected into HUVECs to knockdown LPAR5 expression. Notably downregulated LPAR5 expression was observed after transfection, and HUVECs transfected with si-LPAR5#2 presented a better interference effect (Figure 1c-d). Therefore, si-LPAR5#2 was used to perform further experiments. Results of CCK-8 assay indicated that cell viability was significantly reduced in ox-LDL challenged HUVECs, which was elevated by the further LPAR5 deletion Figure 1e). Additionally, ox-LDL stimulation resulted in dramatically enhanced contents of TNF-α, IL-1β and IL-6 as comparison to the control group, whereas LPAR5 knockdown markedly decreased the levels of aforementioned inflammatory cytokines induced by ox-LDL (figure 1f-h). Meanwhile, ox-LDL-induced HUVECs showed extreme increase in the contents of ROS and MDA as well decrease in the activities of SOD and GSH-Px when compared to the control group (Figure 1i-l). By contrast, si-LPAR5#2 transfection alleviated the impact of ox-LDL treatment on the levels of ROS, MDA, SOD and GSH-Px relative to the ox-LDL+si-NC group. These results reveal that ox-LDL stimulation triggers the high LPAR5 expression and LPAR5 silencing relieves ox-LDL-induced inflammation and oxidative stress of HUVECs.

## LPAR5 knockdown attenuates the endothelium dysfunction of HUVECs triggered by ox-LDL

**Figure 2.**
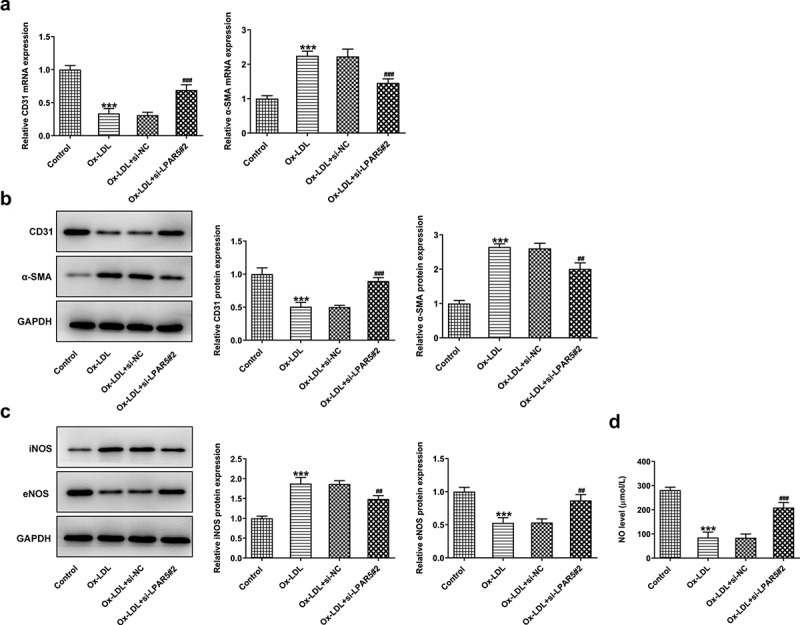
LPAR5 deletion improved the endothelium dysfunction of HUVECs triggered by ox-LDL. (a) CD31 and α-SMA mRNA expression levels after LPAR5 silencing in ox-LDL-induced HUVECs were tested with RT-qPCR. (b) Evaluation of CD31 and α-SMA protein expression with western blotting. (c) Detection of iNOS and eNOS protein expression by means of western blot analysis. (d) The secretion of NO was analyzed with commercially available kit. ***P < 0.001 vs. control; ^##^*P* < 0.01, ^###^*P* < 0.001 vs. ox-LDL+si-NC

Endothelial dysfunction caused by ox-LDL is considered to be a key step in the initiation of AS [17,18]. Subsequently, the effects of LPAR5 deletion on the endothelium dysfunction induced by ox-LDL were evaluated. As displayed in Figure 2a-B, the expression of endothelial marker CD31 was conspicuously downregulated in ox-LDL-exposed HUVECs compared to the control group, accompanied by upregulated expression of mesothelial transition marker a-smooth muscle actin (SMA). On the contrary, as compared to the ox-LDL+si-NC group, LPAR5 loss-of-function apparently increased CD31 expression and decreased a-SMA expression. Consistently, significantly elevated inducible nitric oxide synthase (iNOS) expression and reduced endothelial nitric oxide synthase (eNOS) expression was observed in ox-LDL treatment group, while si-LPAR5#2 reversed these changing trends (Figure 2c). Besides, to elucidate the implication of endothelial protective factor NO in HUVECs challenged with ox-LDL, the concentrations of NO in culture supernatant were examined. It was found that ox-LDL stimulation triggered a notable decrease in NO level when compared to the control group, whereas the further LPAR5 silencing increased NO level. Above findings suggest that LPAR5 knockdown can improve the endothelium dysfunction of HUVECs triggered by ox-LDL.

## LPAR5 silencing inhibits the NLRP3 inflammasome signaling pathway in ox-LDL-exposed HUVECs

**Figure 3.**
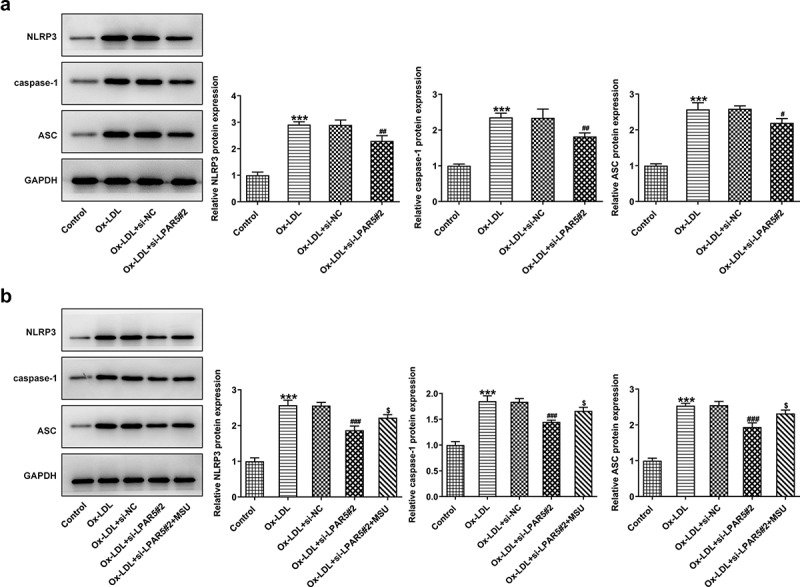
LPAR5 silencing suppressed the NLRP3 inflammasome signaling pathway in ox-LDL-exposed HUVECs. (a) Determination of NLRP3, caspase-1 and ASC expression in ox-LDL stimulated HUVECs after LPAR5 knockdown by western blot analysis. ***P < 0.001 vs. control; ^#^*P* < 0.05, ^##^*P* < 0.01 vs. ox-LDL+si-NC. (b) Test for NLRP3, caspase-1 and ASC expression in ox-LDL stimulated HUVECs after LPAR5 knockdown in the absence or presence of MSU using western blot analysis. ***P < 0.001 vs. control; ^#^*P* < 0.05; ^###^*P* < 0.001 vs. ox-LDL+si-NC; ^$^*P* < 0.05 vs. ox-LDL+si-LPAR5#2

To study the mechanism of LPAR5 silencing in the protective effects of ox-LDL-induced injury of HUVECs, the expression of proteins related to inflammasomes signaling was determined. As what is observable from Figure 3a, the expression of NLRP3, caspase-1 and apoptosis-related speckle-like protein containing cards (ASC) was remarkably upregulated after ox-LDL stimulation compared with the control group. Conversely, in comparison to the ox-LDL+si-NC group, LPAR5 loss-of-function partially blocked the inflammasome signaling pathway, evidenced by downregulated NLRP3, caspase-1 and ASC expression. Afterward, NLRP3 agonist MSU was adopted for treating cells. It was noticed that MSU addition enhanced the expression levels of NLRP3, caspase-1 and ASC when compared to the ox-LDL+si-LPAR5#2 group (Figure 3b). These observations indicate that LPAR5 silencing represses the NLRP3 inflammasome signaling pathway in ox-LDL-induced HUVECs.gs

## Activation of NLRP3 inflammasome signaling abrogates the cytoprotective effects of LPAR5 deletion on ox-LDL-induced HUVECs injury

**Figure 4.**
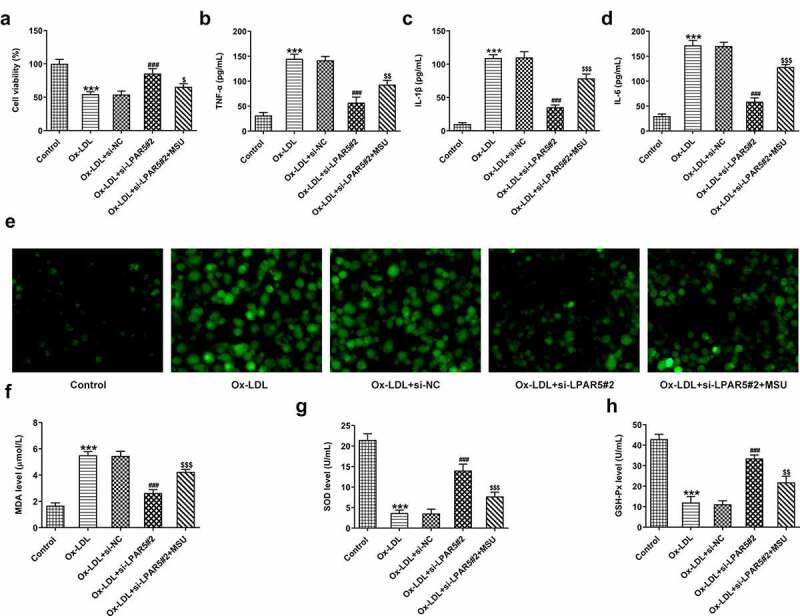
Activation of NLRP3 inflammasome signaling blocked the impacts of LPAR5 silencing on ox-LDL-induced inflammation and oxidative stress of HUVECs. (a) Assessment of cell viability using a CCK-8 kit. The concentrations of (b) TNF-α, (c) IL-1β and (d) IL-6 were examined by ELISA. (e) The determination of intracellular ROS production was tested with a ROS assay kit. Measurement of (f) MDA content as well as (g) SOD and (h) GSH-Px activities using the corresponding commercially available kits. ***P < 0.001 vs. control; ^###^*P* < 0.001 vs. ox-LDL+si-NC; ^$^*P* < 0.05, ^$$^*P* < 0.01, ^$$$^*P* < 0.001 vs. ox-LDL+si-LPAR5#2

**Figure 5.**
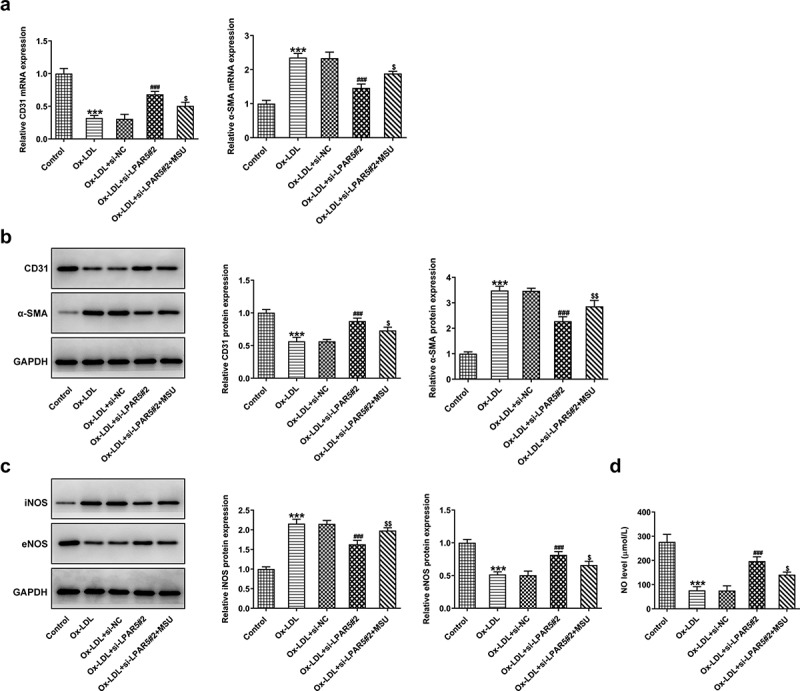
Activation of NLRP3 inflammasome signaling abrogated the effects of LPAR5 deletion on ox-LDL-induced endothelium dysfunction of HUVECs. (a) CD31 and α-SMA mRNA expression levels were evaluated using RT-qPCR. (b) Evaluation of CD31 and α-SMA protein expression with western blotting. (c) Detection of iNOS and eNOS protein expression by means of western blot analysis. (d) The secretion of NO was analyzed with commercially available kit. ***P < 0.001 vs. control; ^###^*P* < 0.001 vs. ox-LDL+si-NC; ^$^*P* < 0.05, ^$$^*P* < 0.01, ^$$$^*P* < 0.001 vs. ox-LDL+si-LPAR5#2

To further clarify whether the protective functions of LPAR5 silencing on the injury of HUVECs induced by ox-LDL was realized through regulating NLRP3 inflammasome signaling, the levels of cell viability, inflammation, oxidative stress and endothelium dysfunction were evaluated after the addition of MSU in ox-LDL-induced HUVECs. As exhibited in Figure 4a, MSU administration notably reduced cell viability relative to the ox-LDL+si-LPAR5#2 group. Concurrently, the secretion of TNF-α, IL-1β and IL-6 in the culture supernatant was obviously elevated after the MSU treatment (Figure 4b-d). As expected, when compared to the ox-LDL+si-LPAR5#2 group, the production of intracellular ROS was significantly increased, coupled with elevated MDA content as well as decreased SOD and GSH-Px activities (Figure 4e-h). Additionally, the expression of CD31 was dramatically downregulated while α-SMA was upregulated in the MSU treated group compared with the ox-LDL+si-LPAR5#2 group (Figure 5a-b). Results of western blot analysis presented in Figure 5c also indicated that MSU led to increased iNOS and decreased eNOS expression. Moreover, a significant reduction in NO level was observed after the addition of MSU in HUVECs induced by ox-LDL with si-LPAR5#2 transfection (Figure 5d). Overall, these data suggest that interference with LPAR5 mitigated injury of HUVECs induced by ox-LDL by suppressing NLRP3 inflammasome signaling.

## Discussion

AS-related cardiovascular diseases are the preeminent cause of death worldwide [19]. It is well known that endothelial damage induced by ox-LDL is considered to be a key contributor to the pathogenesis and progression of AS. Therefore, inhibiting ox-LDL triggered endothelial injury may be a new and effective method for the treatment of AS. Herein, the present study for the first time demonstrated that LPAR5 was highly expressive in HUVECs under ox-LDL condition. Interestingly, our findings suggested that interference with LPAR5 could ameliorate ox-LDL-induced injury of HUVECs by inactivating NLRP3 inflammasome signaling.

A growing body of literature has emphasized the important role of inflammation in the complex pathological process of AS, and AS is now considered to be a complex chronic inflammatory vascular disease [20]. It has been fully proven that ox-LDL can trigger inflammatory response in endothelial cells by elevating the secretion of pro-inflammatory factors, including TNF-α, IL-1β, and IL-6 [21]. Additionally, endothelial cells are easily affected by free radicals and lipid peroxidation, which makes oxidative stress the important pathological cause of endothelial damage [22]. Oxidative stress is thought to be an increase in the biological activity of ROS relative to antioxidant defense [23]. MDA, the end product of lipid peroxidation, can be enhanced when HUVECs exposed to ox-LDL. On the contrary, the antioxidant defense systems are destroyed due to the reduced activities of antioxidant enzyme including SOD and GSH-Px [24,25]. Several previous studies have revealed that LPAR5 participates in the development of AS. For instance, upregulation of LPAR5 expression can transactivate TGFBR1, thereby resulting in inner membrane lipid retention and inducing the early onset of AS [9]. Interestingly, LPAR5 is closely associated to the thrombosis after atherosclerotic plaque rupture [10]. This study was the first to explore the role of LPAR5 in the injury of HUVECs triggered by ox-LDL. We demonstrated that ox-LDL led to a conspicuous upregulation in LPAR5 expression, suggesting that LPAR5 is an AS-related gene, which is in line with the aforementioned research. Importantly, we also revealed that LPAR5 deletion protected against ox-LDL-induced inflammation and oxidative stress in HUVECs.

Endothelial dysfunction caused by ox-LDL is considered to be a key step in the initiation of atherosclerosis [17,18]. Endothelial-mesenchymal transformation (EndMT), a complex biological process through which endothelial cells are transformed into mesenchymal cells, is characterized by the loss of specific endothelial markers and the acquisition of mesenchymal markers [26]. Research has proposed that EndMT participates in the progression of atherosclerosis [27–29]. CD31 is a crucial endothelial marker and α-SMA is a mesothelial transition marker [28]. Besides, eNOS is a key regulator of vascular wall homeostasis. NO, an endothelial protective factor, produced in endothelial cells by a constitutively expressed eNOS, and the dysfunction of eNOS activity can reduce the bioavailability of NO, which contributes to atherosclerosis [30,31]. A large amount of evidence confirms that endothelial dysfunction triggered by ox-LDL is related to the reduced eNOS and elevated iNOS [32,33]. This study suggested that ox-LDL stimulation reduced CD31, eNOS and NO levels as well as enhanced α-SMA and iNOS expression, which was reversed by LPAR5 silencing, implicating the improving effects of LPAR5 downregulation on ox-LDL-induced endothelial dysfunction.

To disclose the underlying mechanism of LPAR5 deletion’s effects on HUVECs injury under ox-LDL condition, the expression of molecules implicated in NLRP3 inflammasome signaling was assessed. Mounting evidence supported that the activation of NLRP3 inflammasome signaling contributes to the development of AS [34,35]. When the NLRP3 inflammasome is activated, NLRP3 will oligomerize with the adaptor protein ASC, resulting in the activation of caspase-1, subsequently causing the secretion of pro-inflammatory factors in ox-LDL-induced HUVECs [36,37]. It is noteworthy that LPAR5 can activate NLRP3 inflammasome in macrophages and contribute to imiquimod-induced psoriasis-like lesions [14]. Findings in the present study demonstrated that LPAR5 knockdown represses the NLRP3 inflammasome signaling in ox-LDL-exposed HUVECs. The further addition of MSU, a NLRP3 agonist, crippled the cytoprotective effects of LPAR5 deletion on ox-LDL-induced HUVECs injury, suggesting that LPAR5 loss-of-function alleviates injury of HUVECs induced by ox-LDL by suppressing NLRP3 inflammasome signaling.

## Conclusions

Taken together, our findings for the first time revealed the protective effects of LPAR5 deletion on injury of HUVECs induced by ox-LDL, as evidenced by inhibition of inflammation, oxidative stress and endothelium dysfunction. Further investigation demonstrated that the cytoprotective effects of LPAR5 deletion on ox-LDL-induced HUVECs injury was realized by suppressing the NLRP3 inflammasome signaling. Findings in this study may serve as an introductory work for future in vivo and in vitro research on the therapeutic value of LPAR5 for AS.

## Data Availability

The datasets used and analyzed are available from the corresponding author on reasonable request.
